# Extraskeletal Mesenchymal Chondrosarcoma of the Uterus

**DOI:** 10.3390/diagnostics12030643

**Published:** 2022-03-05

**Authors:** Yurimi Lee, Sangjoon Choi, Hyun-Soo Kim

**Affiliations:** 1 Department of Pathology and Translational Genomics, Samsung Medical Center, Sungkyunkwan University School of Medicine, Seoul 06351, Korea; yrm.lee@samsung.com; 2 Department of Pathology, Chungnam National University School of Medicine, Daejeon 35015, Korea

**Keywords:** uterus, extraskeletal mesenchymal chondrosarcoma

## Abstract

Mesenchymal chondrosarcoma is an uncommon malignant mesenchymal tumor with an aggressive behavior. Diagnoses of mesenchymal chondrosarcoma are established based on histomorphological, immunohistochemical, and molecular findings. Only one case of extraskeletal mesenchymal chondrosarcoma (EMC) of the uterus has been reported. This article presents the second case of primary uterine EMC, occurring in a 33-year-old woman. We describe the histological and immunophenotypical features of EMC. Our observations will help pathologists and clinicians perform accurate histological diagnoses of uterine EMC and plan appropriate treatment strategies for this rare tumor.

Mesenchymal chondrosarcoma (MC) is an uncommon type of malignant soft tissue tumor, representing fewer than 10% of all chondrosarcoma cases [1]. MC is classified as a high-grade sarcoma with a high risk of metastasis, resulting in a poor prognosis if treated insufficiently [2,3,4]. The diagnosis of MC is established on the basis of histomorphology, in combination with immunohistochemical and molecular findings. Approximately 30% of MCs arise from extraskeletal sites [5,6,7]. These cases are referred to as extraskeletal MCs (EMCs). The most commonly involved sites in EMC are the head and neck, followed by the lower extremities. However, there have been rare reports on EMCs involving various soft tissue and visceral locations [2,3,4,7,8,9]. The female genital tract is a rare site of origin for EMC. Only one case of uterine EMC has been identified, from a thorough literature search [10]. This report presents the second case of primary uterine EMC, occurring in a 33-year-old woman, and describes the clinical, histological, immunophenotypical, and molecular features of uterine EMC.

A 33-year-old woman presented with an abdominal mass. Abdominopelvic magnetic resonance imaging revealed a well-circumscribed, solid mass in the right uterine wall (Figure 1). Her medical and gynecological histories were unremarkable. Degenerated uterine leiomyoma or leiomyosarcoma was suspected. She underwent a total hysterectomy (Figure 1).

Immunostaining was performed using an automated immunostainer (BOND-MAX, Leica Biosystems, Buffalo Grove, IL, USA) [11,12,13,14,15,16,17,18,19]. Table 1 summarizes the panel of antibodies used for the differential diagnosis. Fluorescence in situ hybridization (FISH) for *SS18-SSX* fusion and *SYT-SSX* reverse transcriptase–polymerase chain reaction (RT-PCR) assays were performed. We also performed next-generation sequencing (NGS)-based RNA sequencing to detect relevant gene fusions [19,20].

Representative photomicrographs showing histological features are shown in Figure 2. Microscopic examination revealed the biphasic morphology of undifferentiated mesenchymal and cartilaginous components. Representative photomicrographs showing immunophenotypes are shown in Figure 3. The undifferentiated cells were positive for Bcl-2 and CD99, whereas the cartilages were positive for S100. The lack of a history of EMC in another location, as well as the histological and immunostaining results, supported the diagnosis of primary uterine EMC. The presence of hyaline cartilages and the hemangiopericytoma (HPC)-like vascular pattern made Ewing sarcoma less likely. Synovial sarcoma exhibits HPC-like vasculature [21,22,23]; however, both the FISH for the *SS18*-*SSX* fusion and *SYT*-*SSX* RT-PCR assay were negative. The absence of an immunoreaction towards pan-cytokeratin, desmin, STAT6, CD34, hormone receptors, CD10, and cyclin D1 ruled out uterine carcinosarcoma, leiomyosarcoma, solitary fibrous tumor, and endometrial stromal sarcoma. NGS analysis revealed the fusion of *HEY1-NCOA2*, one of the desirable diagnostic criteria for MC. No *SS18-SSX*, *EWS-FLI1*, or *EWS-ERG* fusion was identified. A final pathological diagnosis of primary uterine EMC was made.

The patient received postoperative whole-pelvic radiation therapy. She is currently alive without evidence of recurrent disease three months after treatment.

Our literature search revealed 18 cases of uterine chondrosarcoma, with some of them showing both mesenchymal and cartilaginous components [10]. However, based on their described clinical and histological features, we classified 17 of the 18 cases as either primary chondrosarcomas, carcinosarcomas, or myxoid chondrosarcomas. Finally, we concluded that uterine EMC reported by Suzuki et al. [10] is the only case showing histologic features compatible with EMC, confirmed by the molecular test. Table 2 summarizes the clinicopathological characteristics of two uterine EMC cases, which share similar gross, histological, and genetic features.

The clinical course of MC is frequently protracted and relentless, requiring a long-term follow-up. Some MC patients have developed distant metastases even after 20 years [2,3,4]. In the only previous case of uterine EMC [10], the patient experienced distant metastasis 52 months after surgery. A longer follow-up period would have been better for comparison and specification of the clinical course of uterine EMC. Nevertheless, our study could provide valuable information on the clinicopathological and genetic features of uterine EMC, and help pathologists to not misdiagnose this rare sarcoma as other tumors.

In summary, we have presented the second case of EMC of the uterus. We noted a biphasic histomorphology of EMC, characterized by undifferentiated small round or spindle tumor cells and islands of hyaline cartilage. An HPC-like staghorn vascular pattern was frequently observed. Positive immunoreactivities for Bcl-2 and CD99 in the undifferentiated component and for S100 in the cartilaginous component, together with the detection of *HEY1-NCOA2* fusion, confirmed the diagnosis of EMC. We anticipate that our comprehensive clinicopathological, immunohistochemical, and molecular analyses will develop better understanding of this unique tumor and help pathologists to perform accurate diagnoses.

## Figures and Tables

**Figure 1:**
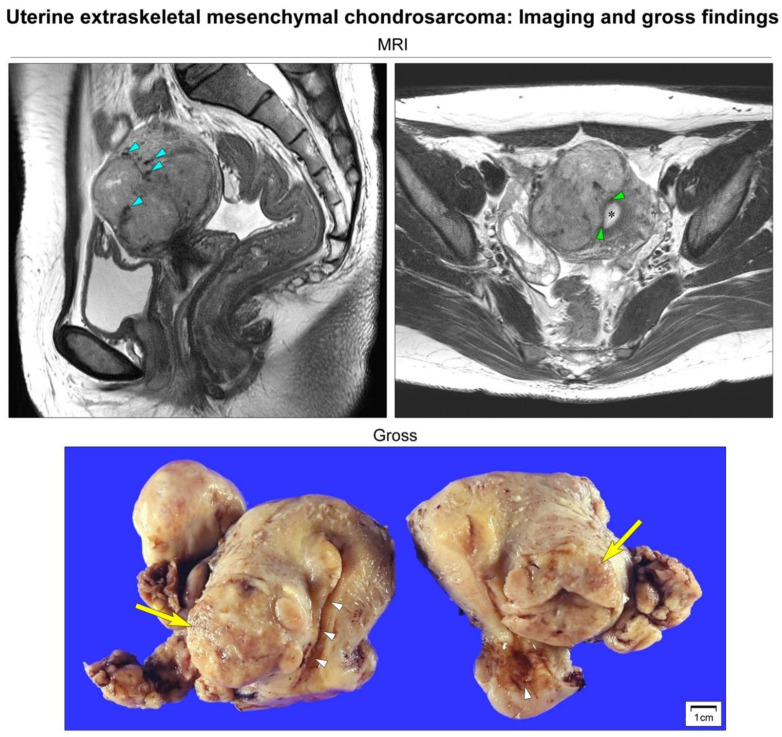
Imaging and gross findings. Abdominopelvic magnetic resonance imaging (MRI) reveals a well-circumscribed, multilobulated, solid mass measuring 6 cm located on the right side of the uterine corpus. T2-weighted sagittal imaging reveals an intramural uterine mass containing punctate, irregular-shaped, hypointense areas of calcification (blue arrowheads). T2-weighted axial imaging reveals that the mass is not connected to the endometrium (black asterisk). The endomyometrial junction (green arrowheads) is intact. Based on the preoperative impression of degenerated uterine leiomyoma or leiomyosarcoma, the patient underwent a total hysterectomy. Grossly, a lobulated, tan-white, rubbery mass (yellow arrows) appears to be confined within the right lateral wall of the uterus. The endocervical and endometrial mucosa (white arrowheads) are unremarkable.

**Figure 2:**
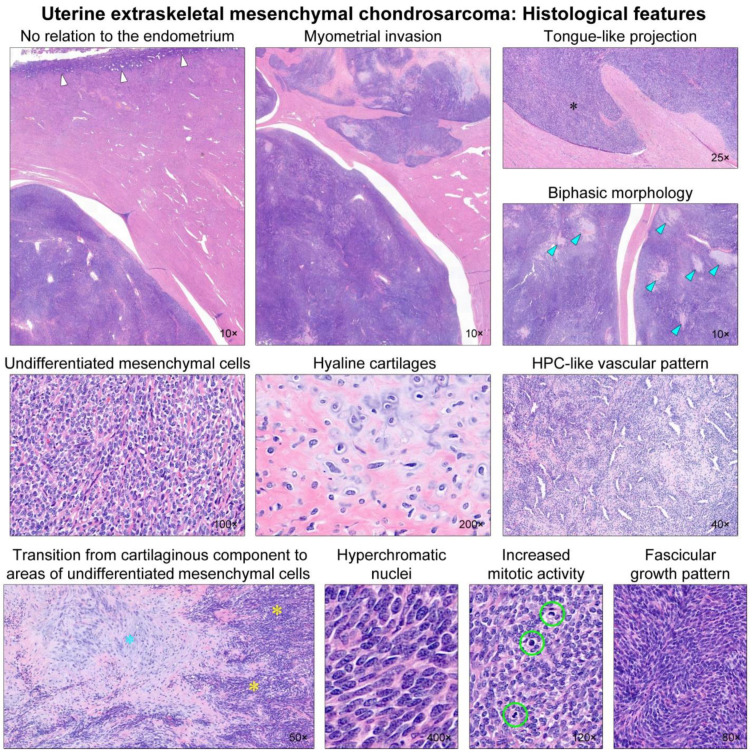
Histological findings. Low-power magnification reveals that the uterine tumor has no relation to the endometrium (white arrows). The tumor permeates into the myometrium with well-delineated margins. Foci of myometrial invasion appear as tongue-like projections (black asterisk), resembling low-grade endometrial stromal sarcoma (LG-ESS). The tumor displays a biphasic morphology, with a so-called ‘white clouds in blue sky’ appearance. Several microscopic islands (blue arrowheads) of a chondroid matrix are scattered randomly within the hypercellular blue areas. The hypercellular component consists of undifferentiated mesenchymal cells, whereas the chondroid component shows hyaline cartilages. Hemangiopericytoma (HPC)-like vascular pattern is occasionally noted, but spiral arterioles resembling LG-ESS are absent. Note a transition from cartilaginous tissue (blue asterisk) to areas of undifferentiated mesenchymal cells (yellow asterisks). High-power magnification reveals that the undifferentiated mesenchymal component displays round-to-polygonal tumor cells with stromal collagen deposition. They possess hyperchromatic, oval-to-spindle-shaped nuclei with evenly dispersed chromatin. The cytoplasm is scant. Brisk mitotic activity (up to 16 per 10 high-power fields; green circles) is observed. Some areas show a fascicular growth pattern with little intervening stroma.

**Figure 3:**
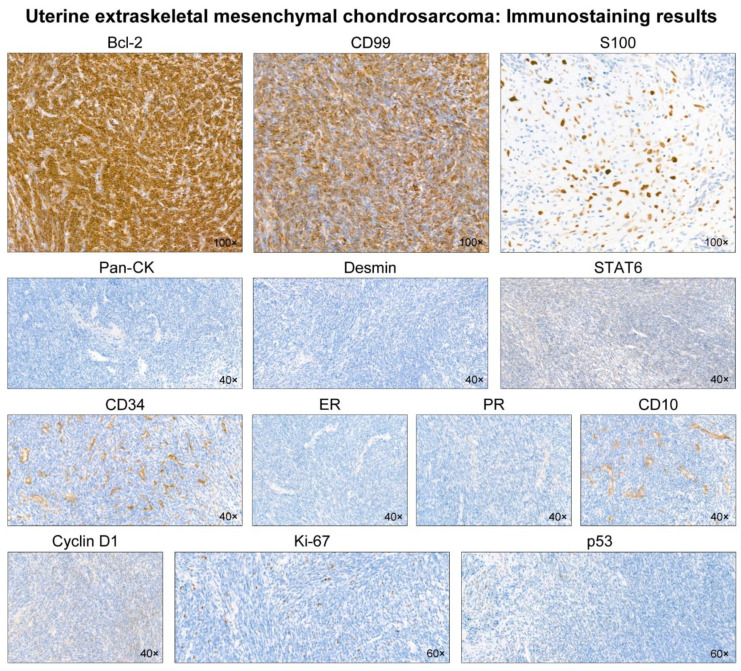
Immunostaining results. The undifferentiated mesenchymal cells display diffuse and strong immunoreactivities for Bcl-2 and CD99. S100 reacts strongly with the nuclei of cartilaginous cells. In contrast, the tumor is negative for pan-cytokeratin (pan-CK), desmin, STAT6, CD34, estrogen receptor (ER), progesterone receptor (PR), CD10, and cyclin D1. The Ki-67 labeling index is low (<10%). p53 immunostaining reveals scattered p53-positive cells with variable staining intensities, indicating a wild-type expression pattern.

**Table 1:** Antibodies used.

Antibody	Clone	Company	Dilution
Bcl-2	124	Dako (Agilent Technologies, Santa Clara, CA, USA)	1:200
CD10	56C6	Novocastra (Leica Biosystems, Buffalo Grove, IL, USA)	1:100
CD34	QBEnd-10	Dako (Agilent Technologies, Santa Clara, CA, USA)	1:400
CD99	PCB1	Novocastra (Leica Biosystems, Buffalo Grove, IL, USA)	1:50
Pan-CK	AE1/AE3	Dako (Agilent Technologies, Santa Clara, CA, USA)	1:500
Cyclin D1	P2D11F11	Novocastra (Leica Biosystems, Buffalo Grove, IL, USA)	1:50
Desmin	D33	Dako (Agilent Technologies, Santa Clara, CA, USA)	1:200
ER	6F11	Novocastra (Leica Biosystems, Buffalo Grove, IL, USA)	1:300
PR	MIB1	Novocastra (Leica Biosystems, Buffalo Grove, IL, USA)	1:1200
Ki-67	DO7	Dako (Agilent Technologies, Santa Clara, CA, USA)	1:200
p53	16	Novocastra (Leica Biosystems, Buffalo Grove, IL, USA)	1:800
S100	Polyclonal	Dako (Agilent Technologies, Santa Clara, CA, USA)	1:5000
STAT6	EP325	Cell Marque (Rocklin, CA, USA)	1:100

**Table 2:** Summary of clinicopathological characteristics of previously reported cases of uterine extraskeletal mesenchymal chondrosarcoma arising in the uterus.

Case	1	2
Author (year published)	Suzuki et al. (2014) [10]	Lee et al. (2022) (present case)
Age of patient	69 years	33 years
Presenting symptom	Lower abdominal distention	Uterine mass on imaging
Previous medical or gynecological history	Absent	Absent
Imaging finding	12 cm well-defined intramural mass	6 cm well-defined intramural mass
Hyaline cartilages	Present	Present
Undifferentiated mesenchymal cells (UMCs)	Present	Present
Hemangiopericytoma-like vascular pattern	Present	Present
Epithelial component	Absent	Absent
Myxoid component	Absent	Absent
Bcl-2	Not applicable	Positive (in UMCs)
CD10	Not applicable	Negative
CD34	Negative	Negative
CD99	Negative	Positive (in UMCs)
Pan-cytokeratin	Focal positive	Negative
Cyclin D1	Not applicable	Negative
Desmin	Negative	Negative
ER	Not applicable	Negative
PR	Not applicable	Negative
Ki-67	Not applicable	Low (<10%)
p53	Not applicable	Wild-type
S100	Not applicable	Positive (in cartilages)
SOX9	Positive (in UMCs)	NA
*HEY1-NCOA2* fusion	Detected	Detected
*SS18-SSX* fusion	Not applicable	Not detected
*EWS-FLI1* fusion	Not applicable	Not detected
*EWS-ERG* fusion	Not applicable	Not detected
Primary treatment	Total hysterectomy	Total hysterectomy
Post-operative treatment	None	Whole-pelvic radiation therapy
Recurrence (location)	Present (bone metastasis)	Absent
Disease-free survival	52 months	3 months

## Data Availability

Not applicable.
